# Directed evolution to improve the catalytic efficiency of urate oxidase from *Bacillus subtilis*

**DOI:** 10.1371/journal.pone.0177877

**Published:** 2017-05-22

**Authors:** Wenjie Li, Shouteng Xu, Biao Zhang, Yelin Zhu, Yan Hua, Xin Kong, Lianhong Sun, Jiong Hong

**Affiliations:** School of Life Sciences, University of Science and Technology of China, Hefei, Anhui, P. R. China; Russian Academy of Medical Sciences, RUSSIAN FEDERATION

## Abstract

Urate oxidase is a key enzyme in purine metabolism and catalyzes the oxidation of uric acid to allantoin. It is used to treat hyperuricemia and gout, and also in a diagnostic kit. In this study, error-prone polymerase chain reaction and staggered extension process was used to generate a mutant urate oxidase with improved enzyme activity from *Bacillus subtilis*. After several rounds of mutagenesis and screening, two mutants 6E9 and 8E279 were obtained which exhibited 2.99 and 3.43 times higher catalytic efficiency, respectively. They also exhibited lower optimal reaction temperature and higher thermo-stability. D44V, Q268R and K285Q were identified as the three most beneficial amino acid substitutions introduced by site-directed mutagenesis. D44V/Q268R, which was obtained through random combination of the three mutants, displayed the highest catalytic activity. The *K*_m,_ *k*_cat_/*K*_m_ and enzyme activity of D44V/Q268R increased by 68%, 83% and 129% respectively, compared with that of wild-type urate oxidase. Structural modeling indicated that mutations far from the active site can have significant effects on activity. For many of them, the underlying mechanisms are still difficult to explain from the static structural model. We also compared the effects of the same set of single point mutations on the wild type and on the final mutant. The results indicate strong effects of epistasis, which may imply that the mutations affect catalysis through influences on protein dynamics besides equilibrium structures.

## Introduction

**Fig 1:**
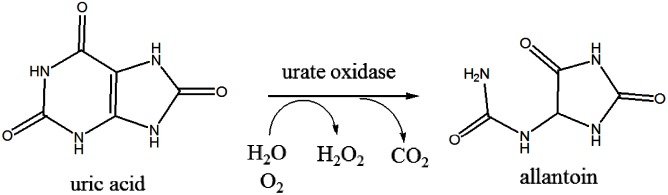
Degradation of uric acid to allantoin.

Urate oxidase, which is also known as uricase, is an important enzyme in purine degradation. It catalyzes uric acid degradation and produces allantoin (Fig 1), which is much more soluble than uric acid [1]. Urate oxidase is present in various organisms such as animals, plants, fungi, yeasts, and bacteria [2]. However, during the evolutionary process, urate oxidase activity seems to have been lost in some higher primates including humans [3, 4]. In these species, the end product of purine metabolism is uric acid rather than allantoin [1].

Uric acid exists in the human body mainly as free acid and urate salt, both of which are insoluble in water. The accumulation of uric acid in the body can result in hyperuricemia. In some cases, its crystallization and precipitation lead to gout [1]. During chemotherapy of leukemia and lymphoma, the excretion of uric acid increased sharply because of the degradation of nucleic acid from malignant cells. The excessive uric acid can obstruct the renal tubules and cause acute renal failure (“tumor lysis syndrome”) [5].

Allopurinol is a drug used to treat gout. It inhibits xanthine oxidase and blocks the formation of uric acid. However, it increases the precursors of uric acid, hypoxanthine and xanthine, which increase the burden on the kidneys. Direct injection of urate oxidase can rapidly degrade uric acid without the accumulation of any additional intermediate products [6]. Urate oxidase coupled with the 4-aminoantipyrine peroxidase system, is also used as a reagent in the clinical diagnostic kit [7].

For clinical applications, it is highly desirable to increase the activity of urate oxidase. Hence, it is necessary to improve the expression and activity of the enzyme by all possible means [8]. There have been reports of urate oxidase purification from fungi, yeast, and bacteria. Large-scale production from these organisms is difficult because of the low expression level and low stability of the enzyme. These difficulties have been overcome by heterologous expression of urate oxidase. Rasburicase, a clinical drug, is a recombinant urate oxidase from *Aspergillus flavus* [5]. Urate oxidase from *Candida utilis* and *Pseudomonas aeruginosa* has also been heterologous expressed [7, 9]. Directed evolution is a powerful tool to improve the catalytic activity of an enzyme. In addition, the mutants obtained by directed evolution often provide some information about the catalytic mechanism.

In this study, we conducted several rounds of mutagenesis coupled with staggered extension process and screened the mutants in *Escherichia coli*, to improve the catalytic activity of *Bacillus subtilis* urate oxidase (BSUO). Several mutants with improved activities were obtained and characterized. To explore the sequence structure-function relationship of these mutants, a series of single point mutations were constructed and analyzed. A homology model using *Bacillus* sp. TB-90 urate oxidase (PDB: 5ayj) as template (its sequence identity to BSUO is 66.78%) has been constructed to guide our discussions.

## Materials and methods

### Chemicals

Uric acid, ampicillin, isopropyl β-D-l-thiogalactopyranoside, lysozyme and all media supplements were purchased from Sangon Biotechnology (Shanghai, China). L-arabinose was purchased from Sigma (Darmstadt, Germany). Restriction endonucleases were purchased from Fermentas (Burlington, Canada). T4 DNA ligase and *Prime STAR* DNA polymerase were purchased from TAKARA (Dalian, China). *Taq* DNA polymerase and deoxy nucleotide triphosphate (dNTP) mix were obtained from Promega (Madison, USA). Oligonucleotides were synthesized by Sangon Biotechnology. A Wizard SV Gel and PCR Clean-Up System (Promega, Madison, WI, USA) was used to isolated DNA fragments from agarose gel. Thermo scientific spectra multicolor broad range protein ladder was purchased from Fermentas (Burlington, Canada).

### Strains, plasmids, and media

*E*. *coli* BL21 (DE3) was used as the host strain for gene expression. Plasmid pBAD/myc-His-A was used for cloning and expression. Luria-Bertani (LB) medium [1% (w/v) tryptone, 0.5% (w/v) yeast extract, and 1% (w/v) NaCl] was used to cultivate *E*. *coli*. L-arabinose (0.2%) was added to the medium for inducing expression.

Gene of urate oxidase from *B*. *subtilis* ATCC 23857 was available in our lab. PCR amplification was performed using pBAD upstream primer and pBAD downstream primer (Table 1). The amplified fragment was inserted into *Nco* I and *Hind* III sites of pBAD vector and transformed into *E*. *coli* BL21 (DE3) cells. The constructed pBAD vector containing urate oxidase gene was designated pBAD-UO.

**Table 1:** Oligonucleotides used in this study.

primer	Sequence(5’-3’)	Products
pBAD upstream primer	CGCAACTCTCTACTGTTTCTC	BSUO
pBAD downstream primer	GCCAAGCTGGAGACCGTTTAA	BSUO
D44-saturation-forward	GTCGGCGTTNNNGTGACATGCGAAATTG	7D44
D44-saturation-reverse	CAATTTCGCATGTCACNNNAACGCCGAC	7D44
E279-saturation-forward	GATACGGTTGTCNNNGAAATCCCGGGC	8E279
E279-saturation-reverse	GCCCGGGATTTCNNNGACAACCGTATC	8E279
T4I-F	CCATGGATAAAAGAA**T**CATGTCTTATGGCAA	T4I
T4I-R	TTGCCATAAGACATG**A**TTCTTTTATCCATGG	T4I
T4N-F	CCATGGATAAAAGAA**A**CATGTCTTATGGCAA	T4N
T4N-R	TTGCCATAAGACATG**T**TTCTTTTATCCATGG	T4N
D44V-F	CCGTTGTCGGCGTTG**T**TGTGACATGCGAAAT	D44V
D44V-R	ATTTCGCATGTCACA**A**CAACGCCGACAACGG	D44V
D44N-F	ACCGTTGTCGGCGTT**A**ATGTGACATGCGAAA	D44N
D44N-R	TTTCGCATGTCACAT**T**AACGCCGACAACGGT	D44N
L100F-F	AGCTCACCGATTTTT**T**GATACCTATTCTCAT	L100F
L100F-R	ATGAGAATAGGTATC**A**AAAAATCGGTGAGCT	L100F
K127E-F	CCTGCATACGAGGAG**G**AAGAGCTCAAGCACA	K127E
K127E-R	TGTGCTTGAGCTCTT**C**CTCCTCGTATGCAGG	K127E
E128G-F	CATACGAGGAGAAAG**G**GCTCAGCACAAGCCG	E128G
E128G-R	CGGCTTGTGCTGAGC**C**CTTTCTCCTCGTATG	E128G
R137G-F	AGCCGCCTCGTCTTC**G**GAAGATCGCGTAATG	R137G
R137G-R	CATTACGCGATCTTC**C**GAAGACGAGGCGGCT	R137G
F182Y-F	ACTCCTTCGTCGGCT**A**CATCCGGGACGAATA	F182Y
F182Y-R	TATTCGTCCCGGATG**T**AGCCGACGAAGGAGT	F182Y
S217T-F	AATGACTCATACGCT**A**CTGATCCAGCACGCT	S217T
S217T-R	AGCGTGCTGGATCAG**T**AGCGTATGAGTCATT	S217T
D264V-F	TCCCGCAGCTCACTG**T**TGTCAGCTTCCAATC	D264V
D264V-R	GATTGGAAGCTGACA**A**CAGTGAGCTGCGGG	D264V
Q268R-F	CTGATGTCAGCTTCC**G**ATCTCAAAATCACAC	Q268R
Q268R-R	GTGTGATTTTGAGAT**C**GGAAGCTGACATCAG	Q268R
E279K-F	TGGGATACGGTTGTC**A**AAGAAATCCCGGGCT	E279K
E279K-R	AGCCCGGGATTTCTT**T**GACAACCGTATCCCA	E279K
K285Q-F	GAAATCCCGGGCTCA**C**AAGGAAAAGTCTACA	K285Q
K285Q-R	TGTAGACTTTTCCTT**G**TGAGCCCGGGATTTC	K285Q
K285I-F	AAATCCCGGGCTCAA**T**AGGAAAAGTCTACAC	K285I
K285I-R	GTGTAGACTTTTCCT**A**TTGAGCCCGGGATTT	K285I
H300R-F	CATACGGTTTCCAAC**G**TTTTACCGTGACAAG	H300R
H300R-R	CTTGTCACGGTAAAA**C**GTTGGAAACCGTATG	H300R
C319F-F	AAGCCGCTGAAAAAT**T**TCGGAGCCTGAAAGC	C319F
C319F-R	GCTTTCAGGCTCCGA**A**ATTTTTCAGCGGCTT	C319F
8E-I4T-F	CCATGGATAAAAGAA**C**CATGTCTTATGGCAA	8E-I4T
8E-I4T-R	TTGCCATAAGACATG**G**TTCTTTTATCCATGG	8E-I4T
8E-Y182F-F	ACTCCTTCGTCGGCT**T**CATCCGGGACGAATA	8E-Y182F
8E-Y182F-R	TATTCGTCCCGGATG**A**AGCCGACGAAGGAGT	8E-Y182F
8E-E127K-F	CCTGCATACGAGGAG**A**AAGAGCTCAGCACAA	8E-E127K
8E-E127K-R	TTGTGCTGAGCTCTT**T**CTCCTCGTATGCAGG	8E-E127K
8E-V44D-F	ACCGTTGTCGGCGTT**G**ATGTGACATGCGAAA	8E-V44D
8E-V44D-R	TTTCGCATGTCACAT**C**AACGCCGACAACGGT	8E-V44D
8E-Q285K-F	GAAATCCCGGGCTCA**A**AAGGAAAAGTCTACA	8E-Q285K
8E-Q285K-R	TGTAGACTTTTCCTT**T**TGAGCCCGGGATTTC	8E-Q285K

Underlined bases in bold font are substitutions.

### Error-prone PCR and library construction

**Fig 2:**
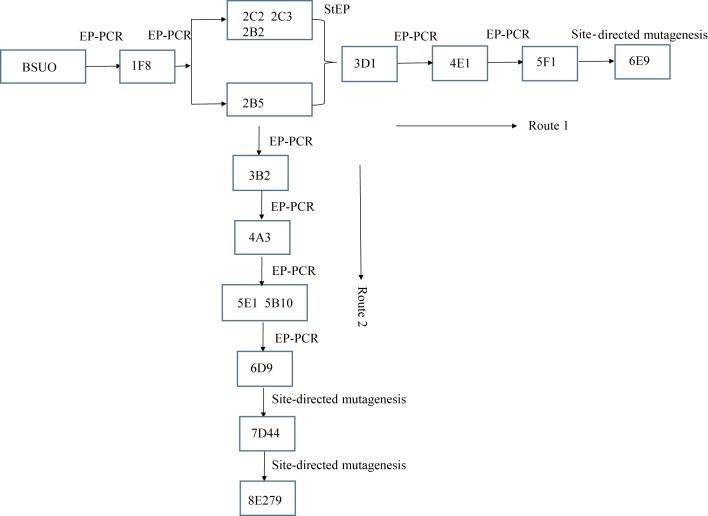
The screening strategy and directed evolutionary history of the mutants.

Random mutagenesis library was generated by error-prone PCR (EP-PCR) [10]. The urate oxidase gene library containing random mutations was amplified using pBAD upstream primer and pBAD downstream primer (Table 1) with *Taq* DNA polymerase. The reaction mixture contained 0.2 mM each of dATP and dGTP, 1.0 mM each of dCTP and dTTP, 7 mM MgCl_2_ and 0.15 mM MnCl_2._ The plasmid pBAD-UO was used as template for the first round. The program was 30 cycles of 30 s at 94°C, 30 s at 50°C and 1 min at 72°C. The PCR product was digested with *Nco* I/ *Hind* III and ligated into plasmid pBAD/myc-His-A. The ligation products were transformed into *E*. *coli* BL21 (DE3), and around 6000 transformants in each cycle were recovered. All clones from the library were subjected to downstream screening assay. The best mutant was picked as the template for the next round screening (Fig 2).

### Staggered extension process

Staggered extension process (StEP) was performed according to the previously described method [11]. The mutants from the above process were used as templates in a PCR-modified staggered extension process using pBAD upstream primer and pBAD downstream primer (Table 1). StEP conditions were 0.5 μl template DNA, 10 pmol of each primer, 0.2 mM each dNTP, 1 × *Taq* buffer, 25 mM Mg^2+^ and 0.5 U *Taq* polymerase. The program was 5 min at 94°C, 80 cycles of 30 s at 94°C and 5 s at 55°C. The resultant 1 kb DNA fragment was digested with *Nco* I/ *Hind* III and ligated into plasmid pBAD/myc-His-A. The ligation products were transformed into *E*. *coli* BL21 (DE3).

### Library screening

Single clones of strain BL21 (DE3) from the urate oxidase random mutagenesis library, harboring mutation were cultivated in 0.2ml LB medium supplemented with 100 μg/ml ampicillin in 96-well deep plates [12]. The plates were incubated at 37°C, with shaking at 250 rpm for 12 h. Twenty microliters of the overnight culture was inoculated into a new 96-well deep plate containing 0.1 ml fresh LB medium and induced with 0.2% L-arabinose (L-ara). After 4 h of induction at 37°C, with shaking at 250 rpm, cells were harvested by centrifugation (4000×*g*, 10 min, 4°C). The cells were subjected to three cycles of freezing at -80°C and thawing and then were resuspended in 300 μl lysis buffer [50 mM Tris-HCl (Tris-Cl), 0.15 mg/ml lysozyme, pH 8.0], and incubated at 37°C for 20 min. After centrifugation (4000×*g*, 10 min), the cell debris was removed and the supernatant was used for subsequent enzyme activity assay.

During the screening process, urate oxidase activity was measured using 96-well plate. Eighty microliters of the crude enzyme of urate oxidase mutant was added into 120 μl of uric acid solution. Uric acid has strong absorption at 293 nm and the molar absorption coefficient is 1.15×10^4^. The absorbance at 293 nm was measured for 5 min at 30°C. Mutants with improved activity were selected for further analysis.

### Site-directed mutagenesis

Site-directed mutagenesis was performed using a PCR-based method [13]. Primers containing appropriate base substitutions are listed in Table 1. PCR was carried out with 1μl template plasmid (pBAD-UO), 0.5 μl *Prime STAR* DNA polymerase, and 1μl of the primer pairs in a final volume of 50 μl. PCR products were digested with *Dpn* I at 37°C for 8 h and then transformed into competent *E*. *coli* BL21 (DE3) cells. The mutations were confirmed by sequencing.

### Enzyme purification and activity assays

BSUO and mutants were expressed with a 6×His tag at the carboxyl terminus in *E*. *coli* BL21 (DE3) cells. A single clone was inoculated into 10 ml LB in a tube and cultured overnight at 37°C with shaking at 250 rpm. The culture was then transferred into a 250 ml conical flask containing 100 ml fresh LB medium containing 100 μg/ml ampicillin and induced with 0.2% L-ara. After 4 h of induction at 37°C, with shaking at 250 rpm, cells were harvested by centrifugation (8000×*g*, 10 min, 4°C) and then disrupted by ultrasonication in 50 mM Tris-Cl buffer (pH 8.5). The urate oxidase enzyme with 6×His tag was purified by a HisTrap^TM^ HP with AKTA purifier system (GE Healthcare, Piscataway, NJ, USA). Purified proteins were analyzed by sodium dodecyl sulfate polyacrylamide gel electrophoresis. Protein concentration was determined using the Bradford assay with bovine serum albumin as a standard [14].

Urate oxidase enzyme activity was assayed in a 4 ml reaction system. Purified enzyme (400 μl) was added to reaction mixture consisting of 600 μl 0.4 mM uric acid (pH 8.5) and 3 ml 50 mM Tris-Cl (pH 8.5). The decrease of uric acid was determined by measuring the absorbance at 293 nm for 7 min at 28°C. One unit of enzyme activity was defined as the amount of enzyme necessary to transform 1μmol of uric acid into allantoin in 1 min at 30°C and pH 8.5.

### Determination of kinetic parameters

To determine the apparent *K*_m_ for the decomposition of uric acid, the enzyme activity was determined in a reaction mixture containing 50 mM Tris-Cl (pH 8.5), 400 μl purified enzyme, and a variable concentration (5, 10, 15, 20, 25, 30, 35 mM) of uric acid at 28°C. Nonlinear regression was used for fitting the data to the Michaelis-Menten equation for determining the kinetic constants [1, 15].

### Effect of temperature and pH on urate oxidase activity and stability

To determine optimal reaction temperature, enzyme activity was measured at 20°C, 25°C, 30°C, 35°C, 40°C, 45°C and 50°C. The thermo-stability of the enzyme was evaluated by incubating the enzyme at 0°C, 20°C, 25°C, 30°C, 35°C, 40°C, 45°C and 50°C for 15 min and assaying the residual activity under standard assay conditions [1].

The optimal pH of the enzyme was determined by measuring relative activity at various pH values (Sodium acetate-acetic acid buffer was used for pH 4.0 and 5.0; disodium hydrogen phosphate—sodium dihydrogen phosphate buffer was used for pH 6.0 and 7.0; Tris-Cl buffer was used for pH 8.0 and 9.0; and sodium dihydrogen phosphate-sodium hydroxide buffer was used for pH 10.0 and 11.0). The concentration of each buffer was 50 mM. The pH stability was also determined by incubating the enzymes at specified pH values at 4°C for 18 h. The residual activities were calculated based on the urate oxidase of non-incubated enzyme [1].

### Molecular modeling studies

Swiss-Model was used to construct homology models. The *Bacillus* sp. TB-90 urate oxidase (PDB: 5ayj) which has been determined at 2.05 Å resolution without ligand was used as the modeling template. It has the highest sequence identity to BSUO (66.78%), so the resultant modeling structure was credible. PyMol was used to analyze the structure of the mutants. The structures of BSUO and all the mutants were constructed using these tools. The structure of the mutant enzyme was compared with that of wild type urate oxidase.

## Results

### Successful cloning and expression of urate oxidase in *E*. *coli* BL21 (DE3)

A 975 bp DNA fragment encoding BSUO was amplified by PCR and cloned into pBAD/myc-His-A. The constructed vector containing BSUO gene designated pBAD-UO was expressed in *E*. *coli* BL21 (DE3) cells. BSUO was purified by Ni-chelating affinity chromatography and analyzed by denaturing gel electrophoresis. The sodium dodecyl sulfate polyacrylamide gel electrophoresis showed a single band of ~38 kDa which is in agreement with the molecular weight calculated from the amino acid sequence (S1 Fig).

### Two variants of urate oxidase with improved activity were obtained through directed evolution

**Fig 3:**
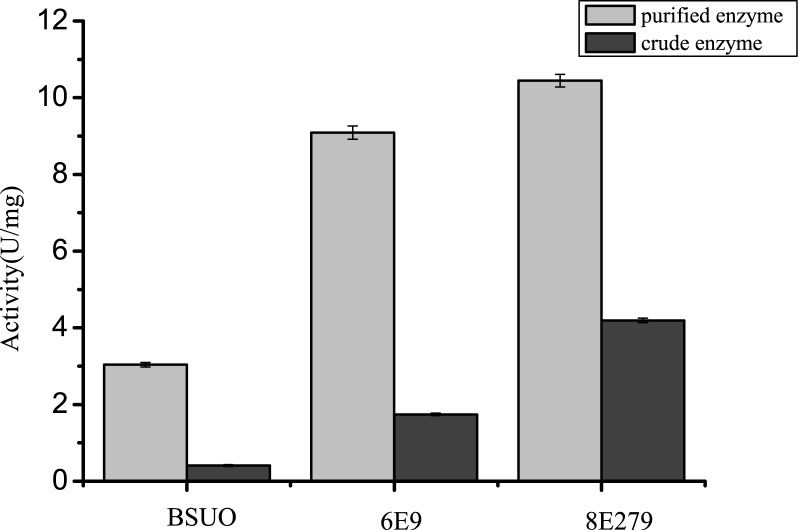
Activities of BSUO and mutants 6E9 & 8E279.

In order to improve the activity of wild type BSUO, random mutagenesis libraries were constructed through error prone PCR (EP-PCR). After two rounds of EP-PCR, we obtained four mutants with similar activities and named them 2B2, 2B5, 2C2 and 2C3. Directed evolution was achieved through two different routes. In the first route, the four mutants were combined through staggered extension process and 3D1 was isolated through screening (Fig 2). We then conducted three more rounds of EP-PCR and finally obtained mutant 6E9. In the second route, four rounds of EP-PCR using 2B5 as the template were conducted which yielded the mutant 6D9. When these two mutants, 6E9 and 6D9 were compared, we found that there were some different mutagenesis sites. Using 6D9 as the template and site-directed saturation mutagenesis, we introduced a mutation at position D44 and obtained mutant 7D44. We then used 7D44 as template to introduce mutation at position E279 and obtained mutant 8E279 (Fig 2). The enzyme activities of mutants 6E9 and 8E279 were analyzed (Fig 3). The crude extracts had activities of 1.74 U/mg and 4.19 U/mg which were 9.12 and 13.79 folds higher, respectively, compared to that of BSUO crude enzyme. Purified 6E9 and 8E279 displayed a specific activity of 9.09 U/mg and 10.44 U/mg respectively, which were 2.99 and 3.43 folds higher than that of wild type BSUO (Fig 3). These data suggested that the mutants not only showed improved catalytic efficiency but also showed higher expression.

### The mutants were more thermo-stable than wild type BSUO

**Fig 4:**
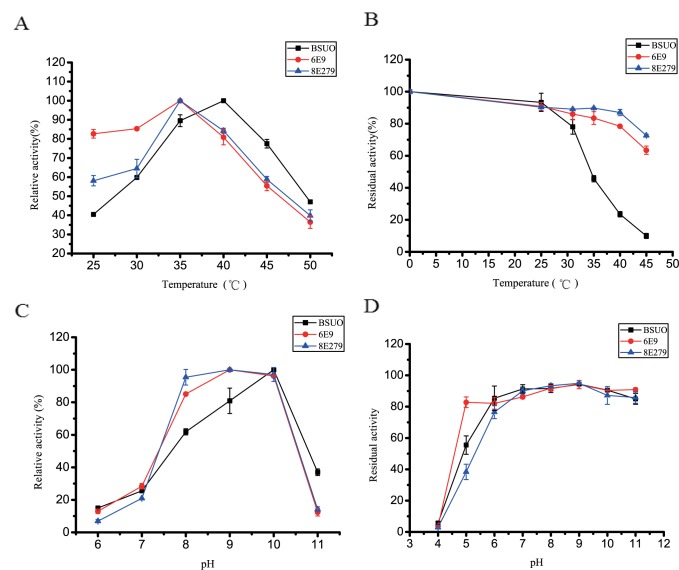
Characterization of purified urate oxidase. (A) Optimum temperature determination. (B) Thermal-stability. (C) Optimum pH. (D) pH stability

To investigate the effects of temperature and pH on the catalytic efficiency, purified wild type BSUO and mutants 6E9 and 8E279 were characterized. The optimum temperature was found to be 40°C for wild type BSUO and 35°C for the mutants. The activity of 6E9 and 8E279 decreased to 81% and 84% respectively at 40°C (Fig 4A). However the thermo-stability of mutants increased. After incubation at 40°C for 15 min, the activity of wild type BSUO decreased to 23%. Almost no activity was detected when the temperature changed to 45°C. 6E9 and 8E279 displayed 63% and 73% residual activity after incubation at 45°C for 15 min (Fig 4B).

### Optimum pH of mutants was different from that of wild type BSUO

The mutants had a different optimum pH compared to that of wild type BSUO. The highest activity for wild type BSUO was observed at pH 10.0, decreased to 62% at pH 8.0 and to 80% at pH 9.0. 6E9 and 8E279 exhibited highest activity at pH 9.0, which decreased to 85 to 95% at pH 8.0 and to 96 to 97% at pH 10.0. The pH stability of the wild type BSUO was same as the mutants. Enzymes were more stable in alkaline conditions (Fig 4C and 4D).

### Site-directed mutagenesis to identify beneficial amino acid substitutions

**Fig 5:**
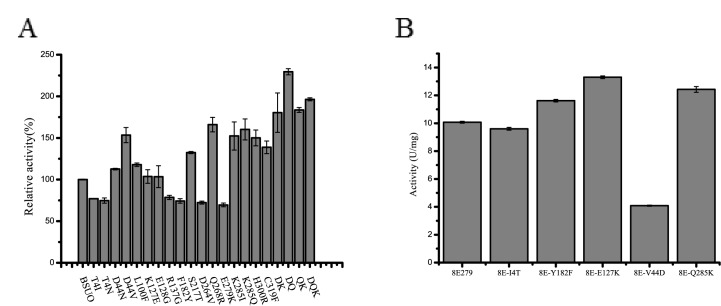
Activity of purified wild-type BSUO and mutants. (A) Relative activity of BSUO and mutants. T4I, T4N, D44N, D44V, L100F, K127E, E128G, R137G, F182Y, S217T, D264V, Q268R, E279K, K285I, K285Q, H300R and C319F are single point mutants; DK and DQ are double mutants from D44V; QK is double mutant from Q268R; DQK is triple mutant from DQ. (B) Activity of purified 8E279 and reverse mutants. 8E-I4T, 8E-Y182F, 8E-E127K, 8E-V44D and 8E-Q285K represent mutants which have reverse mutations.

Site-directed mutagenesis was performed to understand the effect of the various mutations in 6E9 and 8E279. Seventeen single point mutations of BSUO, T4I, T4N, D44V, D44N, L100F, K127E, E128G, R137G, F182Y, S217T, D264V, Q268R, E279K, K285Q, K285I, H300R, and C319F were constructed. The mutants D44V, S217T, Q268R, K285I, K285Q, H300R and C319F showed higher activity demonstrating an improvement of 1.53, 1.32, 1.66, 1.51, 1.60, 1.45 and 1.39 times respectively compared to that of wild type BSUO (Fig 5A). The activity of the mutants D44N, L100F, K127E and E128G was similar to that of wild type BSUO, whereas the activity of the remaining six mutants was lower (Fig 5A). We also evaluated the combination of D44V, Q268R, and K285Q, which were the best among the 17 single point mutants (Fig 5A). The activities of mutants D44V/Q268R, D44V/K285Q and Q268R/K285Q were 6.79 U/mg, 5.44 U/mg, 5.43 U/mg, which were 2.29, 1.80, 1.83 times greater than that of wild type BSUO. The activity of all three mutants D44V/Q268R, Q268R/K285Q and D44V/K285Q was improved compared to that of single point mutants D44V, Q268R and K285Q. We speculated that any two of the three amino acid substitutions D44V, Q268R and K285Q may have a synergistic effect. D44V/Q268R has the highest catalytic activity indicating that D44V and Q268R may have the best synergistic effect. However, the triple mutant, D44V/Q268R/K285Q showed a specific activity of 5.8 U/mg which was only 1.96 folds higher compared to that of wild type BSUO.

In order to further understand the effect of single mutation on catalytic efficiency, we used 8E279 as template to do reverse mutagenesis. Mutations T4I, F182Y, K127E, D44V and K285Q which have different effect on catalytic efficiency were chosen (Fig 5A). Fig 5B showed that 8E-I4T had similar activity to 8E279 rather than higher activity. Mutant 8E-Y182F showed higher activity. Mutant 8E-E127K and 8E-Q285K surprisingly had higher activity than 8E279. Mutant 8E-V44D had a lower activity which is agree with the effect on D44V. All of the effects on reversed mutants except for 8E-V44D and 8E-Y182F were significantly different from the effects on wild type. These results suggested that these mutations may have strong epistasis effects [16].

### Comparative kinetic analyses of mutants

To investigate the catalytic mechanism, all the mutants from the screening step and the wild type BSUO were purified and kinetic parameters of some mutants were measured (Table 2). The *K*_m_ of 6E9 and 8E279 increased by 20% and 50% respectively. The *k*_cat_ of 6E9 and 8E279 were 2 and 3.68 times higher than that of wild type BSUO. And the *k*_cat_/*K*_m_ of 6E9 and 8E279 were 2.42 and 3.09 times greater than that of wild type BSUO (Table 2). The increase of the *k*_cat_ and *K*_m_ of 6E9 and 8E279 suggested that the amino acid substitutions in 6E9 and 8E279 may improve the catalytic efficiency but reduce the substrate affinity.

**Table 2:** Kinetic characteristics of BSUO and mutants.

	*K*_m_ (μmol/L)	*k*_cat_ (s^-1^)	*k*_cat_*/K*_m_ (s^-1^/μmol·L^-1^)	Location
BSUO	27.14 ±2.11	7.07 ±0.27	0.26 ±0.01	—
T4I^a^	34.12 ±1.70	3.23 ±0.12	0.10 ±0.00	S1 ^d^ Part II
T4N^a^	37.58 ±0.24	6.30 ±0.09	0.17 ±0.00	S1 ^d^ Part II
R137G^a^	36.91 ±0.45	6.75 ±0.05	0.18 ±0.00	S4´^d^ Part II
F182Y^a^	47.76 ±0.02	3.79 ±0.02	0.08 ±0.00	Loop between S5 ^d^ and S6 ^d^ Part II
D264V^a^	28.45 ±1.05	5.84 ±0.09	0.21 ±0.00	S7 ^d^ Part II
E279K^a^	31.65 ±2.20	5.10 ±0.18	0.16 ±0.01	Loop between S7´^d^ and S8´^d^ Part II
D44N^b^	35.68 ±0.56	10.01 ±0.03	0.28 ±0.01	S2 ^d^ Part III
K127E^b^	29.08 ±0.20	6.41 ±0.00	0.22 ±0.00	Loop between S3´^d^ and S4´^d^ Part II
E128G^b^	30.98 ±2.77	7.20 ±0.50	0.23 ±0.01	S4´^d^ Part II
K285I^b^	47.71 ±4.07	12.83 ±0.59	0.27 ±0.01	Loop between S7´^d^ and S8´^d^ Part II
D44V^c^	27.03 ±1.37	11.61 ±0.31	0.43 ±0.01	S2 ^d^ Part III
L100F^c^	27.13 ±0.58	9.67 ±0.05	0.37 ±0.01	Helix H2 ^d^ Part III
S217T^c^	31.45 ±1.07	10.61 ±0.49	0.34 ±0.01	Helix h2 ^d^ Part III
Q268R^c^	33.40 ±8.06	15.80 ±2.63	0.48 ±0.04	S7 ^d^ Part II
K285Q^c^	33.40 ±3.09	14.10 ±0.40	0.42 ±0.00	Loop between S7´^d^ and S8´^d^ Part II
H300R^c^	30.27 ±0.41	11.32 ±0.17	0.37 ±0.00	S8 ^d^ Part II
C319F^c^	27.27 ±0.94	9.50 ±0.26	0.35 ±0.01	—
DQ	45.50 ±1.09	21.67 ±0.20	0.48 ±0.01	—
DK	37.66 ±0.25	17.48 ±0.37	0.46 ±0.01	—
QK	46.12 ±3.95	21.51 ±1.80	0.47 ±0.00	—
DQK	35.76 ±2.64	15.43 ±0.81	0.43 ±0.01	—
6E9	32.70 ±1.61	20.60 ±0.58	0.63 ±0.01	—
8E279	41.06 ±2.80	33.04 ±1.53	0.81 ±0.02	—
8E-I4T	47.41 ±2.57	39.51 ±1.58	0.83 ±0.01	—
8E-Y182F	32.72 ±0.52	31.66 ±0.30	0.97 ±0.03	—
8E-E127K	37.07 ±1.10	38.15 ±0.72	1.03 ±0.01	—
8E-V44D	38.30 ±1.86	11.37 ±0.28	0.30 ±0.01	—
8E-Q283K	39.15 ±4.27	40.19 ±3.32	1.03 ±0.03	—

^a^ mutants with lower catalytic efficiency.

^b^ mutants with similar activity.

^c^ mutants with higher catalytic efficiency to wild type.

^d^ the name of each fragment of the structure is according to reported research [17].

T4I, T4N, D44N, D44V, L100F, K127E, E128G, R137G, F182Y, S217T, D264V, Q268R, E279K, K285I, K285Q, H300R and C319F are single point mutants; DQ, DK, QK are the pair-wise combination mutants D44V/Q268R, D44V/K285Q and Q268R/K285Q, respectively. DQK is the triple mutant D44V/Q268R/K285Q.

To analyze the contribution of these substitutions on urate oxidase activity, the catalytic properties of all the single point mutants were characterized and compared with those of wild type BSUO (Table 2). The *k*_cat_/*K*_m_ of T4I, T4N, R137G, F182Y, D264V and E279K were lower than that of wild type BSUO. The *k*_cat_/*K*_m_ of D44N, K127E, E128G and K285I were similar to that of wild type BSUO. As for mutants D44V, L100F, S217T, Q268R, K285Q, H300R and C319F, the *k*_cat_/*K*_m_ of them were higher than that of wild type BSUO. Among these single point mutants, D44V, Q268R and K285Q exhibited the highest catalytic efficiency. The *k*_cat_/*K*_m_ of D44V, Q268R and K285Q were 1.65, 1.83 and 1.62 times higher than that of wild type BUSO.

The *k*_cat_/*K*_m_ of D44V/Q268R, D44V/K285I and Q268R/K285I, which were double mutations, were 1.82, 1.78 and 1.79 times higher than that of wild type BSUO. The *K*_m_ of D44V/Q268R, D44V/K285I and Q268R/K285I increased by 68%, 39% and 70% respectively. However, the *k*_cat_ of D44V/Q268R, D44V/K285I and Q268R/K285I improved 2.07, 1.47 and 2.04 folds higher of wild type BSUO *k*_cat_. Therefore, the catalytic efficiency of these mutants were improved, and the two mutations in the double mutants may have a synergistic effect.

In order to achieve the highest possible degree of improvement in the catalytic activity, a mutant combining all three mutations was constructed and its kinetic parameters were measured. The *K*_m_ of D44V/Q268R/K285I increased by 30%, whereas the *k*_cat_ of D44V/Q268R/K285I was 2.18 folds higher of wild type BSUO *k*_cat_. The *k*_cat_/*K*_m_ improved 1.65 times compared to that of wild type BSUO (Table 2).

### Structure analysis of the urate oxidase

**Fig 6:**
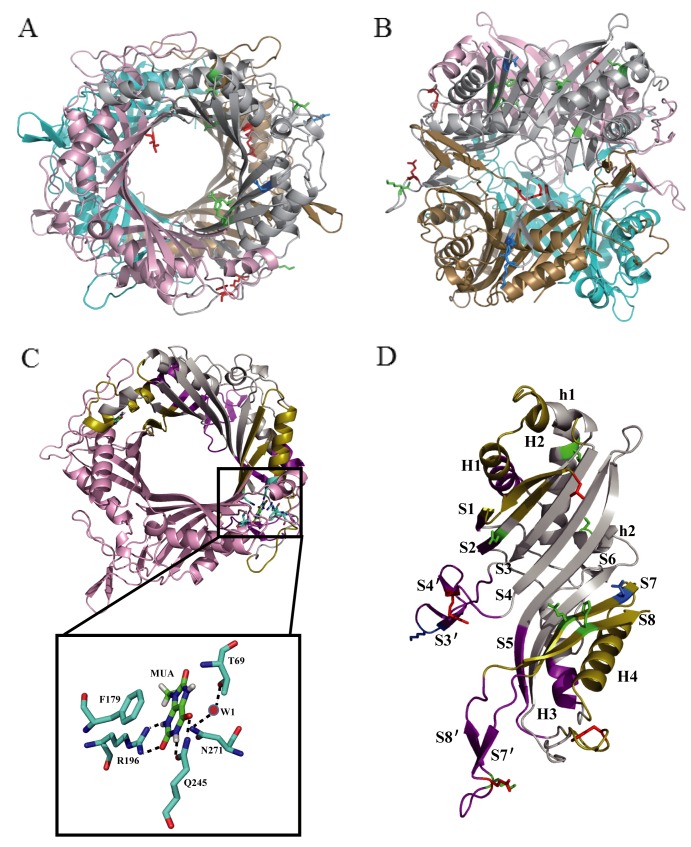
Simulated structure of urate oxidase. (A) Top view of the tetramer. (B) Side view of the tetramer. (C) The dimer. Active sites show in cyan (Part I) and substrate analog MUA shows in green. Water shows in red. (D) The monomer. Part II shows in olive (dimeric interface) and purple (tetrameric interface). Part III shows in gray. Stick in red represents the mutations which have lower catalytic efficiency. Stick in marine represents the mutations exhibiting similar activity to wild type BSUO. Stick in green represents the mutations which have higher catalytic efficiency.

To understand the structure-function relationship of the mutants, we modeled and overlaid the structures of mutants and BSUO. The putative structure of wild type BSUO exhibits four identical subunits (Fig 6A and 6B). The monomer is composed of two successive domains, which are called the T-fold domain for tunneling-fold domain [17]. Each domain has four antiparallel *β*-sheets and a pair of α helices layered on the concave side of the sheet. The concatenation of two T-fold domains gives rise to an antiparallel *β*-sheets of eight sequential strands with the helices layered on the concave side of the sheet [17]. There is an inter subunit antiparallel *β*-sheet between residues near the N-terminus of one subunit and those near the C-terminus of the other subunit of a homodimer, which encloses a tightly closed tunnel [8, 18]. The functional tetramer results from the union of two dimers stacked face- to-face [19].

The active site located at the interface of the dimers is highly conserved. It is composed of two subunits, one of which contributes T69 and the other contributes F179, R196, Q245 and N271. Uric acid interacts with the enzyme through hydrogen bonds with R196 and Q245, an aromatic pi-stacking with F179. And there is a catalytic water located above the uric acid interacts with the enzyme through hydrogen bonds with N271 and T69 from another subunit (Fig 6C). The function of the inside tunnel is not clear. It is speculated that the tunnel may allow flexibility to fulfill cooperativity between the subunits [17].

All the mutations except C319F were marked in the monomer structure (Fig 6D). We divided the monomer structure into three parts according to their location and function: part Ι was the active site, all the active residues were located in this part. Part II was the interaction interface including the dimeric interface and tetrameric interface. All the other structures constituted part III (Table 2 and Fig 6D). This part including the β strands which composed the tunnel and α helices exposed to the outside of the protein. T4I/T4N locates at the N terminal of strand S1. D44N/D44V locates at strand S2. K285I/K285Q locates at the loop between strand S7´and S8´. E128G and R137G locate at strand S4´. D264V and Q268R locate at the same strand S7. H300R locates at strand S8. K127E, F182Y and E279K locate at the loop between S3´ and S4´, loop between strand S5 and S6 and loop between strand S7´and S8´. L100F and S217T locate at helix H2 and h2, respectively.

## Discussion

Urate oxidase is an enzyme involved in the purine degradation pathway. In the process of evolution, two independent nonsense mutations at position 33 and 187 in the urate oxidase gene of humans resulted in the inactivation of the enzyme [3, 20]. Therefore there is a high level of uric acid in human body which reduces the level of free radicals and the possibility of cancer [4, 21]. Abnormalities in the metabolism of uric acid leads to diseases like hyperuricemia, gout, renal failure and so on. Urate oxidase can efficiently reduce the level of uric acid and degrade uric acid deposits in human body. It is also part of a diagnostic kit used to determinate the concentration of uric acid in blood, which is clinically significant [22, 23].

It is very important to choose the starting material for directed evolution. For expressing the recombinant enzyme in *E*. *coli*, urate oxidase from microorganisms is preferable to mammalian enzymes [8]. For clinical applications, it is important for the enzyme to be stable under physiological conditions. Urate oxidase from *Aspergillus flavus* has a favorable thermo-stability at 4°C but exhibits lower activity under physiological conditions [24]. At the same time, the rate of allergic reaction is high due to immunogenicity [25, 26]. BSUO functions optimally at 37°C and has 50% residual activity when incubated at 37°C for 12 h [27]. The thermo-stability of mutants constructed in this study was improved further. BSUO has a high sequence identity of 66% with *Bacillus* sp. TB-90 urate oxidase, which has crystal structure available (PDB: 5ayj). Therefore, it is helpful in examining the structure-function relationship.

Directed evolution can improve the properties of an enzyme including catalytic activity and stability. The incomplete understanding of the catalytic mechanism of BSUO makes rational design difficult. Besides, in rational design, mutations are generally restricted to the amino acids around active site, ignoring other potential beneficial substitutions which are not in the active site. In this study, we conducted directed evolution of BSUO. After several rounds of random mutagenesis and screening, the two best mutants 6E9 and 8E279 were obtained. There were 17 amino acid substitutions in these mutants. Using site-directed mutagenesis, we constructed 17 mutants with single amino acid substitution. The activity and kinetic parameter of these mutants were measured. Among these mutants, D44V, Q268R and K285Q were the best three single point mutants.

**Fig 7:**
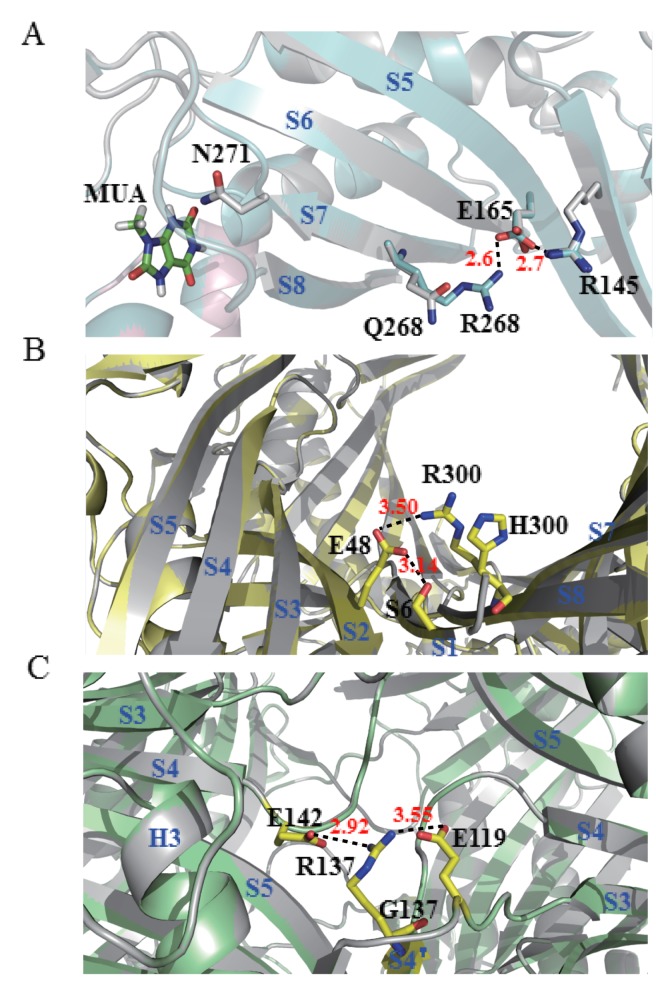
The structure modification of mutants Q268R, H300R and R137G. (A). Superposition of BSUO (represents in gray) and mutant Q268R (represents in cyan). MUA shows in green. In the structure of BUSO, Q268 is too far to interact with E165. When it is substituted by R268, the distance between R268 and E165 shorten to 2.6Å and forms a new ionic bond. (B) Superposition of BSUO (represents in gray) and mutant H300R (represents in paleyellow). When H300 transform into R300, R300 forms a new bond with E48 from neighboring subunit. At the same time, E48 forms a bond with S6 from the same subunit. (C) Superposition of BSUO (represents in gray) and mutant H300R (represents in palegreen). R137 has an ionic bond with E142 of subunit from other dimer and with E119 from same subunit. When it is changed into G137, both interactions are vanish.

In order to investigate the catalytic mechanism of BSUO, we use Swiss-model to build its homologous model. None of these mutations locates in the active site and most mutations locate in part II, only D44V, L100F and S217T locate in part III (Table 2 and Fig 6D). Mutants Q268R, K285Q and H300R locate in part II, and the *k*_cat_/*K*_m_ of them is 1.85, 1.62 and 1.42 folds higher of wild type BSUO (Table 2), respectively. The homologous modeling suggests that the Q268R substitution may form a new ionic bond with E165. And E165 may interact with D145 within the same subunit (Fig 7A). The H300R substitution may form a new ionic bond with E48 of the other subunit from the same dimer (Fig 7B). This interaction can improve the ability of two monomers to form a dimer and further promoted two dimers form a functional tetramer. The kinetic parameter of T4I, R137G, F182Y, D264V and E279K shows these substitutions lead to the decrease of catalytic efficiency. And the affinity of enzyme with substrates also decrease except for D264V. The decrease of *k*_cat_ and the increase of *K*_m_ eventually lead to the decrease of *k*_cat_/*K*_m_ (Table 2). The structure alignment shows that R137 may form an ionic bond with E142 of the subunit from the other dimer. When R137 is changed into G137, this connection between two dimers is vanish (Fig 7C). The *k*_cat_/*K*_m_ of K127E and E128G is similar to that of wild type BSUO (Table 2). All of these mutations which located in part II do not show structure modification except for mutants Q268R, H300R and R137G. Despite the activity of these mutants changed, the mechanism is not clear. The activity change of mutants D44V, L100F and S217T from part III may because of their increase of *k*_cat_. The *k*_cat_ of D44V, L100F and S217T is 1.64, 1.37 and 1.50 times greater than that of wild type BSUO (Table 2). But the structure alignment shows no difference. It is difficult to demonstrate functions of amino acids located far from the active site [12, 28, 29]. The underlying mechanisms are difficult to explain from the static structure model. The mutations may affect catalysis through influences on protein dynamics [30]. Similarly, it has been reported that mutations far from the active site can improve the catalytic activity of other enzymes [31–33].

Some reverse mutations were constructed to further study the catalytic mechanisms. Mutations T4I and F182Y had most significantly decrease of catalytic efficiency in single point mutants because of the decrease of *k*_cat_ and the increase of *K*_m_. When the mutations were reversed, the catalytic efficiency of 8E-I4T and 8E-Y182F should have be improved. The *k*_cat_/*K*_m_ of 8E-Y182F was increased because of the decrease of *K*_m_. Although the *k*_cat_ of 8E-I4T was increased, the *k*_cat_/*K*_m_ of 8E-I4T was similar to 8E279 because of the increase of *K*_m_ (Table 2). The catalytic efficiency of D44V or K285Q increased most significant in single point mutants because of the increase of *k*_cat_. When the mutations were reversed, the catalytic efficiency of 8E-V44D and 8E-Q285K should have be decreased. The *k*_cat_/*K*_m_ of 8E-V44D was decreased because of the decrease of the *k*_cat_. But the *k*_cat_ and *k*_cat_/*K*_m_ of 8E-Q285K were increased (Table 2). Mutation K127E seemed had no effect on catalytic efficiency in single point mutant. When the mutation was reversed, the *k*_cat_ and *k*_cat_/*K*_m_ of 8E-E127K were surprisingly increased (Table 2). The effect of some mutations on reversed mutants, was different from the effect on the wild type. These results means that these mutations may exhibit strong epistasis.

In summary, the mutants 6E9 and 8E279 with high catalytic activities of 9.09 U/mg and 10.44 U/mg were obtained via directed evolution. The catalytic activity of these mutants was 3 folds higher of wild type BSUO. The mutants also have a lower temperature optimum of 35°C and better thermo-stability. Through site-directed mutagenesis, we constructed a series of mutants. Modeling structures showed that mutations far from the active site were difficult to identify their roles in catalytic efficiency. The results of reverse mutations indicated that these mutations may exhibit strong epistasis. These two observations together may imply that besides affecting equilibrium structures, the mutations may affect catalysis through modulations of protein dynamics.

## Supporting information

S1 Fig SDS-PAGE analysis of purified urate oxidase. (A) M: Protein marker. Lane 1: Crude BSUO. Lane 2: Purified BSUO. Lanes 3–15: purified enzyme of mutants T4I, T4N, D44V, D44N, L100F, K127E, E128G, R137G, F182Y, S217T, D264V, Q268R. (B) M: Protein maker. Lanes 1–9: purified enzyme of mutants E279K, K285Q, K285I, H300R, C319F, DK, DQ, QK, DQK.
(EPS)
